# Case Report: *SATB2*-Associated Syndrome Overlapping With Clinical Mitochondrial Disease Presentation: Report of Two Cases

**DOI:** 10.3389/fgene.2021.692087

**Published:** 2021-06-21

**Authors:** Yuri A. Zarate, Hilary J. Vernon, Katherine A. Bosanko, Praveen K. Ramani, Murat Gokden, Karin Writzl, Marija Meznaric, Tina Vipotnik Vesnaver, Raghu Ramakrishnaiah, Damjan Osredkar

**Affiliations:** ^1^Section of Genetics and Metabolism, University of Arkansas for Medical Sciences, Little Rock, AR, United States; ^2^Department of Genetics, Johns Hopkins University School of Medicine, Baltimore, MD, United States; ^3^Department of Pediatric Neurology, University of Arkansas for Medical Sciences, Little Rock, AR, United States; ^4^Department of Pathology, University of Arkansas for Medical Sciences, Little Rock, AR, United States; ^5^Clinical Institute of Medical Genetics, University Medical Centre Ljubljana, Ljubljana, Slovenia; ^6^Faculty of Medicine, Institute of Anatomy, University of Ljubljana, Ljubljana, Slovenia; ^7^Department of Radiology, University Medical Centre Ljubljana, Ljubljana, Slovenia; ^8^Division of Neuroradiology and Pediatric Radiology, University of Arkansas for Medical Sciences, Little Rock, AR, United States; ^9^Department of Pediatric Neurology, University Children's Hospital, University Medical Centre Ljubljana, Ljubljana, Slovenia

**Keywords:** *SATB2*, glass syndrome, mitochondrial disease, muscle biopsy, *SATB2*-Associated syndrome

## Abstract

*SATB2*-associated syndrome (SAS) is an autosomal dominant neurogenetic multisystemic disorder. We describe two individuals with global developmental delay and hypotonia who underwent an extensive evaluation to rule out an underlying mitochondrial disorder before their eventual diagnosis of SAS. Although the strict application of the clinical mitochondrial disease score only led to the designation of “possible” mitochondrial disorder for these two individuals, other documented abnormalities included nonspecific neuroimaging findings on magnetic resonance imaging and magnetic resonance spectroscopy, decreased complex I activity on muscle biopsy for patient 2, and variation in the size and relative proportion of types of muscle fibers in the muscle biopsies that were aligned with mitochondrial diseases. SAS should be in the differential diagnoses of mitochondrial disorders, and broad-spectrum diagnostic tests such as exome sequencing need to be considered early in the evaluation process of undiagnosed neurodevelopmental disorders.

## Introduction

Mitochondrial diseases are a heterogeneous group of clinical disorders due to deficiency of the respiratory chain. While primary mitochondrial disease (PMD) refers to disorders whose underlying genetic cause directly impairs the composition or function of the electron respiratory chain, secondary mitochondrial dysfunction (SMD), by contrast, refers to any abnormal mitochondrial function other than PMD (Falk, 2010). A broad variety of disorders and pathologic processes can result in SMD including myopathies and muscular dystrophies, other genetic disorders, chromosomal abnormalities, neurodegenerative disorders, inborn errors of metabolism, and autism spectrum disorders (ASDs), among others (Falk, 2010; Niyazov et al., 2016; Frye, 2020).

Phenotypically, whether PMD or SMD, mitochondrial disorders are typically characterized by a broad clinical spectrum often involving multiple organs such as skeletal muscles, brain, heart, liver, kidney, or endocrine glands (Bianchi et al., 2003). With overlapping clinical phenotypes being caused by alterations in several different genes and mutations in the same mitochondrial disease gene leading to different phenotypes, the diagnosis of mitochondrial disease is often challenging. Most diagnostic algorithms include a combination of clinical, biochemical, neuroradiologic, and genetic data (Parikh et al., 2015; Witters et al., 2018).

*SATB2*-associated syndrome (SAS; Glass syndrome, OMIM 612313) is an autosomal dominant neurogenetic multisystemic disorder caused by variants in *SATB2* at 2q33.1 (Zarate et al., 2019). Clinical features of SAS include developmental delay with severe speech delay, hypotonia, palate and dental abnormalities, behavioral difficulties, seizures, and skeletal anomalies (Zarate and Fish, 2017). Individuals with SAS are also often found to have nonspecific abnormalities on neuroimaging such as delayed myelination or abnormal white matter hyperintensities [T2/fluid-attenuated inversion recovery (FLAIR)] (Lewis et al., 2020). Here, we report two individuals with SAS who were extensively examined for a possible mitochondrial disorder before their molecular testing that confirmed an alteration in *SATB2*. Given the described overlapping clinical phenotypes between mitochondrial diseases and SAS, particularly early in life, we conclude that SAS should be considered in the differential diagnosis of individuals undergoing evaluation for mitochondrial dysfunction.

## Materials and Methods

Participants were recruited into the SAS clinical registry through a referral by a treating clinician, a facilitated inquiry by the testing laboratory, direct contact by a caregiver, or via the SAS support group. Individuals with a molecularly confirmed diagnosis of SAS were eligible for the study. Medical records including laboratory results were reviewed.

### Molecular Investigations

#### Patient 1

Initially, targeted exome sequencing (ES) was performed using next-generation sequencing on the isolated DNA sample. The fragmentation and enrichment of the isolated DNA sample were performed according to the protocol Illumina TruSight One, with subsequent sequencing on Illumina MiSeq (Illumina Inc., San Diego, CA). After duplicates were removed, the alignment of reads to UCSC hg19 reference assembly was done using BWA algorithm (v0.6.3), and variant calling was done using GATK framework (v2.8). Proband whole ES was performed as previously described (Bergant et al., 2018). The pathogenic variant was confirmed by Sanger sequencing in the proband. Parental testing was performed on blood-derived DNA by Sanger sequencing. Reanalysis of the initial clinical ES data revealed that the *SATB2* variant was missed because of low coverage. The analysis of the mitochondrial genome was performed using next-generation sequencing of the source DNA sample. Variant calling was performed using HaplotypeCaller in GATK v 2.8. The analysis of variants detected in the mitochondrial genome was performed using MITOMASTER tool [with Cambridge Reference Sequence (rCRS)] and MitoSeek.

#### Patient 2

Trio ES was performed according to standard procedures (Retterer et al., 2016). The entire mitochondrial genome was amplified and sequenced using solid-state sequencing by synthesis process. DNA sequence was assembled and analyzed in comparison to rCRS.

### Biochemical and Histological Investigations

Muscle biopsies were processed by a standard battery of histological, histochemical, and immunohistochemical investigations (Dubowitz and Sewry, 2007). For both individuals, oxidative phosphorylation enzymology activities (complex I, II, III, and IV) were tested in a mitochondrial preparation that was immediately isolated from skeletal muscle with enzymatic activities normalized for citrate synthase activity (Bugiani et al., 2004). For patient 1, analysis for the expression of myosin heavy-chain isoforms with quantitative analysis of muscle fiber diameters and an immunohistochemical analysis for the expression of laminin α2 (antibodies reacting with fragments 80 and 300 kDa of laminin) was also conducted (Sewry, 2000).

## Case Presentations

### Patient 1 (SATB2-214)

The patient was born at 39 weeks' gestation with birth weight of 3.55 kg (66th centile), length of 53 cm (95th centile), and head circumference of 35 cm (66th centile). Prenatally, his mother received escitalopram during the first 8 weeks of gestation. Family history was significant for a maternal uncle with epilepsy. At 2 months, he was noted to have decreased eye contact, and an ophthalmology evaluation showed strabismus with horizontal nystagmus. At 8 months, he was noted to have hypotonia. He walked at 27 months, and by 36 months of age, he displayed global developmental delay and was receiving all therapies. He said his first word at 6 years of age. Psychological testing at 6 years 10 months of age (Bayley scales of infant development III) revealed cognitive development equal to age appropriate achievements of 23 months.

**Figure 1:**
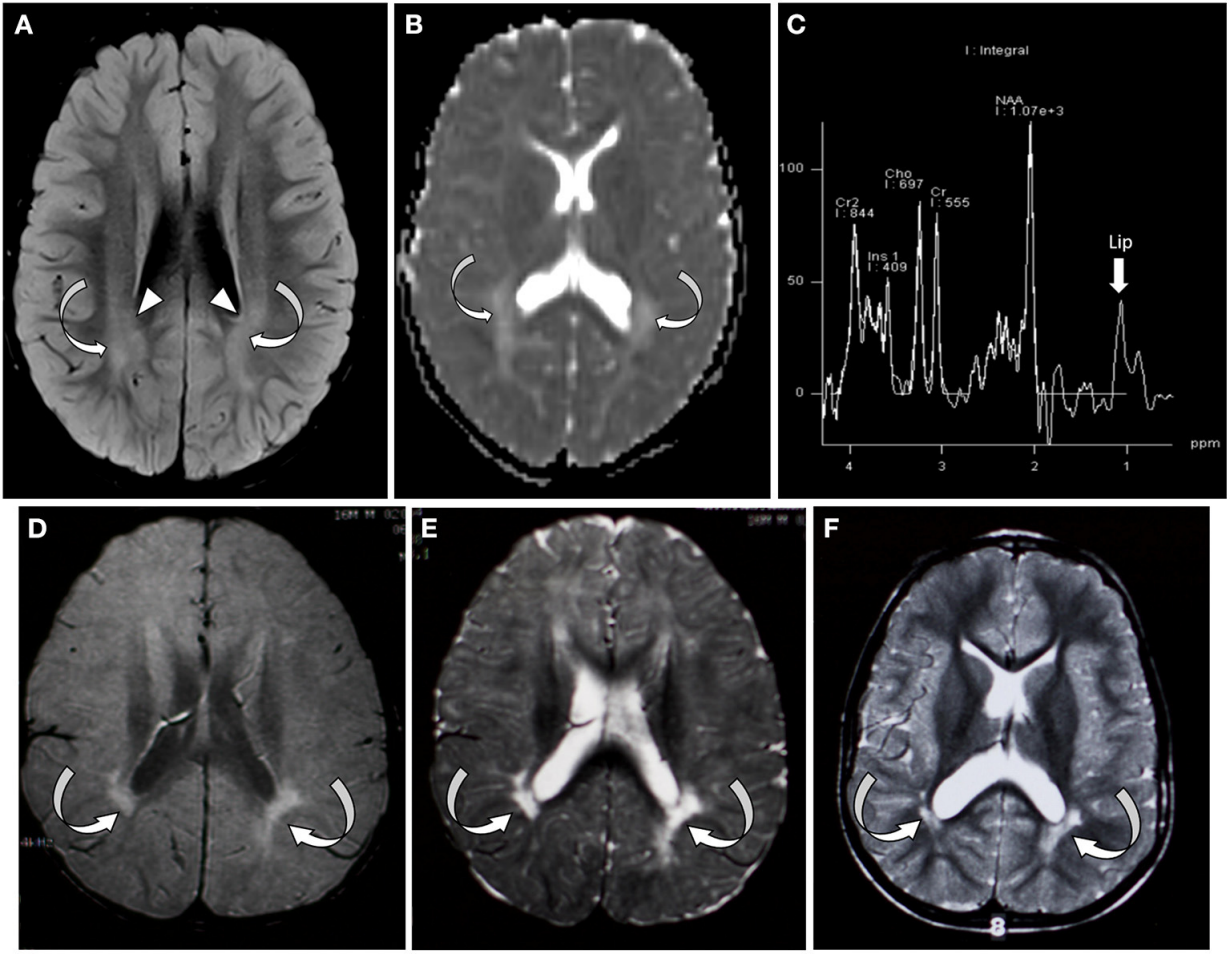
Brain MRI/MRS from patient 1 **(A–C)** and patient 2 **(D–F)**. **(A)** Axial FLAIR sequence at the level of the lateral ventricle and centrum semiovale at 4 years of age showing bilateral symmetric areas of amorphous hyperintensity (curved arrows) within the periventricular white matter. Notice the hyperintensity is seen extending up to the ventricular margin (arrowhead). **(B)** Apparent diffusion coefficient map of the brain through the lateral ventricles showing areas of hyperintensity suggestive of facilitated diffusion (absent diffusion restriction/curved arrows). **(C)** Single-voxel MRI spectroscopy with the voxel placed over the white matter showed slightly elevated lipid peak (arrow). **(D–F)** Axial FLAIR and axial T2 images through the centrum semiovale and ventricles performed at 1, 2, and 4 years of age, respectively. Arrows indicate bilateral periventricular white matter thinning and hyperintensity, predominantly in the periatrial region.

At 8 months, his initial metabolic laboratories were largely unremarkable (Table 1). Brain magnetic resonance imaging (MRI) at 14 months showed patchy areas of higher intensity signal on T2 and FLAIR sequences in the deep white matter bilaterally. Magnetic resonance spectroscopy (MRS) showed various regions with high choline with normal *N*-acetylaspartate (NAA)/creatinine ratio. At 21 months of age, muscle biopsy revealed no morphological abnormalities of the mitochondria and normal respiratory enzyme activity (Supplementary Figure 1). Morphometry showed slight hypotrophy of both types 1 and 2 myofibers, but normal variability coefficients of muscle fiber diameters. Repeat brain MRI at 49 months showed abnormal high signal in the deep white matter temporally, parietally, and occipitally (Figures 1A,B) with a largely unremarkable MRS (reported as subtle peak of lipids and α-glutamate, Figure 1C). At 4 years of age, targeted ES was normal. Sequencing of the mitochondrial genome from muscle- and blood-derived DNA was normal. At 7 years, whole ES revealed a pathogenic variant in *SATB2* (NM_001172509.1), c.1165C>T, and p.(Arg389Cys). The variant was not found in DNA derived from parental lymphocytes.

**Table 1:** Clinical characteristics of two individuals with *SATB2*-associated syndrome.

	**Patient 1 (SATB2-214)**	**Patient 2 (SATB2-79)**
Gender	Male	Male
Mutation (DNA)	c.1165C>T	c.1515delT
Substitution (protein)	p.(Arg389Cys)	p.(Phe505Leufs*41)
Inheritance	*De novo*	*De novo*
Age at last examination	7 years	19 years
**Clinical**		
Developmental delay	Global, severe	Global, severe
Hypotonia	Yes	Yes
Growth retardation	No	Early in infancy
Epilepsy	No	Single febrile seizure
**Testing**		
Brain MRI (age)	Delayed myelination; increased T2 and FLAIR signal in deep white matter (14 and 49 months)	Delayed myelination, increased T2 signal in the periatrial white matter, mild ventricular dilatation, and anterior thinning of corpus callosum (1, 2, and 4 years)
Brain MRS (age)	Slightly elevated lipid peak (49 months)	Slight decrease in N-acetylaspartate (18 months)
Muscle biopsy (age)	Mild hypotrophy of fast and slow twitch muscle fibers; no morphological mitochondrial changes (21 months)	Occasional atrophic appearing type I and neural cell adhesion molecule positive myofibers (4 years)
Respiratory chain findings	Normal	Abnormal; decreased complex I, complex I + III, and complex II + III activity
Plasma amino acids	At 8 months, alanine 1.3 × above normal	Unremarkable
Urine organic acids	Unremarkable	Unremarkable
Urine amino acids	N/A	Unremarkable
Carnitine	Normal	Normal
Lactate	Normal	Normal
Pyruvate	Normal	Normal
Creatine phosphokinase	N/A	Normal
Cerebrospinal fluid lactate	Normal	Normal
Mitochondrial DNA (muscle)	Negative	Negative
Mitochondrial DNA (blood)	Negative	Negative
**Mitochondrial disease criteria (points)**		
Muscular	1 (Motor developmental delay)	1 (Motor developmental delay)
Neurological	2 (Developmental delay, speech delay)	2 (Developmental delay, speech delay)
Multisystem	0	1 (Growth delay early)
Metabolic	0	0
Imaging	0	0
Total points (clinical, metabolic, imaging)	3/8 (Possible)	4/8 (Possible)
Morphology	0	0
Total mitochondrial disease criteria (MDC) score	3/12	4/12

### Patient 2 (SATB2-79)

This individual was previously reported as part of a large cohort study with no detailed clinical information provided (Zarate et al., 2019). He was born at 36 weeks' gestation weighing 2.94 kg (70th centile) and 53.3 cm long (98th centile). Pregnancy was complicated by preterm labor at 28 weeks of age, and family history was noncontributory. He had a significant delay in his motor milestones, sitting by 11 months and walking at 21 months. Speech delay was noticeable during the second year of life but with little progression over time. From early infancy, he was a poor feeder and was diagnosed with failure to thrive necessitating a gastrostomy feeding tube (G-tube), which was placed at 2 years of age. He had a single febrile seizure at 3 years of age. Starting in childhood, the patient exhibited behavioral problems including self-stimulatory behaviors, hoarding, and other obsessive–compulsive behaviors. He was diagnosed with ASD. On his most recent evaluation at 19 years of age, he had severe intellectual disability and intermittent aggressive behaviors. Dysmorphic features included widely spaced incisors, retrognathia, a high-arched palate, bilateral fifth finger clinodactyly, and a left-sided supernumerary nipple. His parents reported limited oral intake, and most nutrition was obtained from G-tube feedings. At 21 years of age, treatment with antipsychotic medication was initiated for aggressive behavior.

**Figure 2:**
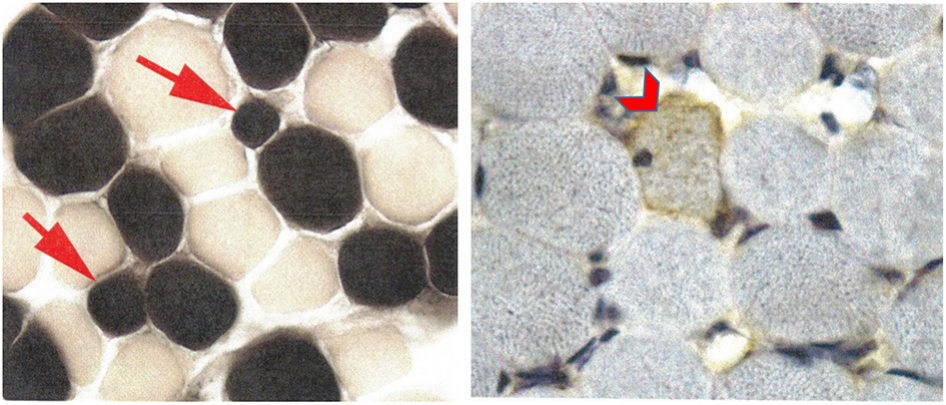
Patient 2 muscle biopsy. ATPase reaction at pH 4.3 shows a few, but not all, type 1 myofibers to be atrophic (arrows) without angulated contours. Neural cell adhesion molecule (NCAM) is weakly positive in a rare myofiber (chevron) without evidence of myopathic or neurogenic changes.

The patient underwent an extensive genetic and metabolic evaluation during childhood (Table 1). Brain MRI scans obtained during childhood showed delayed myelination with increased T2 signal in the periatrial white matter, mild ventricular dilation, and anterior thinning of the corpus callosum (Figures 1D–F). MRS showed a slight decrease in NAA. The patient had a quadriceps muscle biopsy at 4 years 10 months of age, which showed occasional atrophic-appearing type I myofibers (Figure 2A) and occasional neural cell adhesion molecule (NCAM)–positive muscle fibers (Figure 2B). Oxidative phosphorylation enzymology revealed decreased complex I activity, decreased complex I + III activity, and decreased complex II + III activity. Individual testing of complex III and IV showed normal activity. Based on these results, he was diagnosed with oxidative phosphorylation disease with complex I defect. He was treated with various vitamin supplements including coenzyme Q10 and levocarnitine for a few years with no significant improvements noted. Genetic testing at 19 years of age included a normal single-nucleotide polymorphism array, normal transferrin electrophoresis, and normal Angelman/Prader-Willi syndrome methylation testing. Clinical trio-ES revealed a *de novo* pathogenic variant in *SATB2* (NM_015265.3):c.1515delT, p.(Phe505Leufs^*^41). No pathogenic variants were identified in the blood- or muscle-derived mitochondrial DNA. Hearing evaluation, ophthalmologic examination, echocardiogram, and electroencephalogram were normal.

## Discussion and Conclusion

Mitochondrial diseases with respiratory chain dysfunction are characterized by a broad phenotype. While skeletal muscles and brain are the most commonly affected tissues, other organs and tissues such as heart, liver, kidney, and endocrine glands may also be involved (Falk, 2010). Clinical manifestations can include hypotonia, gross and/or fine motor delay, seizures, growth retardation, and behavioral difficulties in the autism-spectrum range that may present at any age (Witters et al., 2018). Similarly, SAS can present with any of these overlapping neurodevelopmental features early in life (Zarate et al., 2018). Indeed, according to a recent review of the SAS phenotype, besides the universal presence of developmental delay, hypotonia, growth retardation, and seizures are present in 59, 31, and 20% of individuals, respectively (Zarate et al., 2019). We here describe two unrelated individuals with SAS who, because of the clinical phenotypic overlap, were investigated for a presumed mitochondrial disease before their final diagnosis was made. Patient 1 had the most commonly reported single-nucleotide variant in SAS (Arg389Cys), whereas patient 2 had a unique truncating variant (Phe505Leufs^*^41). In retrospect, both cases had an overall phenotype that was consistent with SAS. To our knowledge, this is the first report of individuals with SAS that describes detailed muscle histology and enzymology, as well as brain MRS.

The strict application of the mitochondrial disease score (clinical, metabolic, and imaging) led to the designation of “possible” mitochondrial disorder (Witters et al., 2018) for the individuals described in this report. While one individual had documented abnormal respiratory chain complex activity, the other had nonspecific abnormalities with no clear evidence of mitochondrial disease. Some overlapping phenotypic features with mitochondrial disorders are worth mentioning. First, on neuroimaging, as seen in the two individuals from this report, individuals with SAS have nonspecific abnormalities such as delayed myelination (62%) or abnormal white matter hyperintensities (T2/FLAIR) (48%) (Lewis et al., 2020). In mitochondrial disorders, signal abnormalities and hyperintensities are often seen but tend to be bilateral, symmetrical, and with predominant involvement of the basal ganglia and brainstem (Falk, 2010; de Beaurepaire et al., 2018). Second, while neither of the individuals described in this report had a lactate peak, patient 2 did have a slight decrease in NAA on MRS. The most common metabolic brain abnormalities identified in mitochondrial diseases on MRS include a variable decrease in NAA and accumulation of lactate in brain (Bianchi et al., 2003). Third, muscle respiratory chain studies for patient 2 revealed decreased complex I activity. Isolated deficiency of complex I is the most commonly identified biochemical defect in childhood-onset mitochondrial disease (Fassone and Rahman, 2012). Lastly, variation in the size and relative proportion of the type of muscle fibers (along with NCAM expression), as seen in the muscle biopsies of the individuals described here, have been reported in patients with various mitochondrial respiratory chain dysfunctions (Enns et al., 2005).

Accounting for ~0.3% of individuals with unexplained developmental delay/intellectual disability, pathogenic variants in *SATB2* are one of the most common causes of syndromic intellectual disability (Deciphering Developmental Disorders, 2017; Zarate et al., 2017). Mitochondrial diseases, on the other hand, are the most frequent neurometabolic disease with an estimated incidence of ~1/5,000 births (Witters et al., 2018). Therefore, it is possible that an individual with SAS could also have a mitochondrial disease. For the two individuals here described, pathogenic variants in mitochondrial (from muscle and blood) or nuclear (from blood) encoded respiratory chain proteins were ruled out. The subtle phenotypic overlap including the abnormal respiratory chain finding in patient 2 could be an indication of an SMD. It is known that individuals with nonsyndromic ASD or with genetic disorders associated with ASD but not classically associated with mitochondrial disease (such as Down syndrome, Rett syndrome, or Phelan-McDermid syndrome) can have mitochondrial dysfunction and respond to mitochondrial interventions (Niyazov et al., 2016; Frye, 2020). The lack of documented improvement to such treatment in patient 2 could be reflective of the well-known variable response to these interventions, the predominant SAS phenotype, or the absence of a mitochondrial dysfunction amenable to correction.

In summary, we present two individuals with SAS with early-onset neurodevelopmental phenotypes. In addition to some overlapping signs and symptoms also seen in mitochondrial diseases, both individuals had nonspecific findings on neuroimaging and muscle histology that are sometimes seen in mitochondrial disorders. While both individuals were only in the “possible” mitochondrial disorder category, other more distinctive findings such as lactate abnormalities on plasma, cerebrospinal fluid, or MRS could have been missed because of episodic nature of those findings. The possibility of an SMD in SAS would need a larger cohort of individuals evaluated systematically who would undergo dedicated testing to be confirmed. In the interim, we propose that SAS should be in the differential diagnoses of mitochondrial disorders, and broad-spectrum diagnostic tests such as ES should be considered early in the evaluation process of undiagnosed neurodevelopmental disorders.

## Data Availability Statement

The datasets for this article are not publicly available due to concerns regarding participant/patient anonymity. Requests to access the datasets should be directed to the corresponding author.

## Ethics Statement

The studies involving human participants were reviewed and approved by Institutional Review Board of the University of Arkansas for Medical Sciences. Written informed consent to participate in this study was provided by the participants' legal guardian/next of kin. Written informed consent was obtained from the individual(s), and minor(s)' legal guardian/next of kin, for the publication of any potentially identifiable images or data included in this article.

## Author Contributions

YZ analyzed and interpreted the data, prepared, and corrected the manuscript. HV, KB, PR, MG, RR, DO, TV, MM, and KW interpreted the data and were involved in the revision of this manuscript. All authors read and approved the final manuscript.

## Conflict of Interest

The authors declare that the research was conducted in the absence of any commercial or financial relationships that could be construed as a potential conflict of interest.
