# Supplementary material for: Case Report: SATB2-Associated Syndrome Overlapping With Clinical Mitochondrial Disease Presentation: Report of Two Cases
Source: Front Genet. 2021 Jun 21;12:692087. doi: 10.3389/fgene.2021.692087 (PMC8257052; doi:10.3389/fgene.2021.692087)

Supplementary Figure 1. Normal muscle morphology. Hematoxylin and eosin (A), Laminin α2 (80 kDa) (B), Myosin heavy chain-1 (slow) (C), Myosin heavy chain-2a+2x (fast) (D), Modified Gomori trichrome (E), combined cytochrome oxidase and succinic dehydrogenase (F).

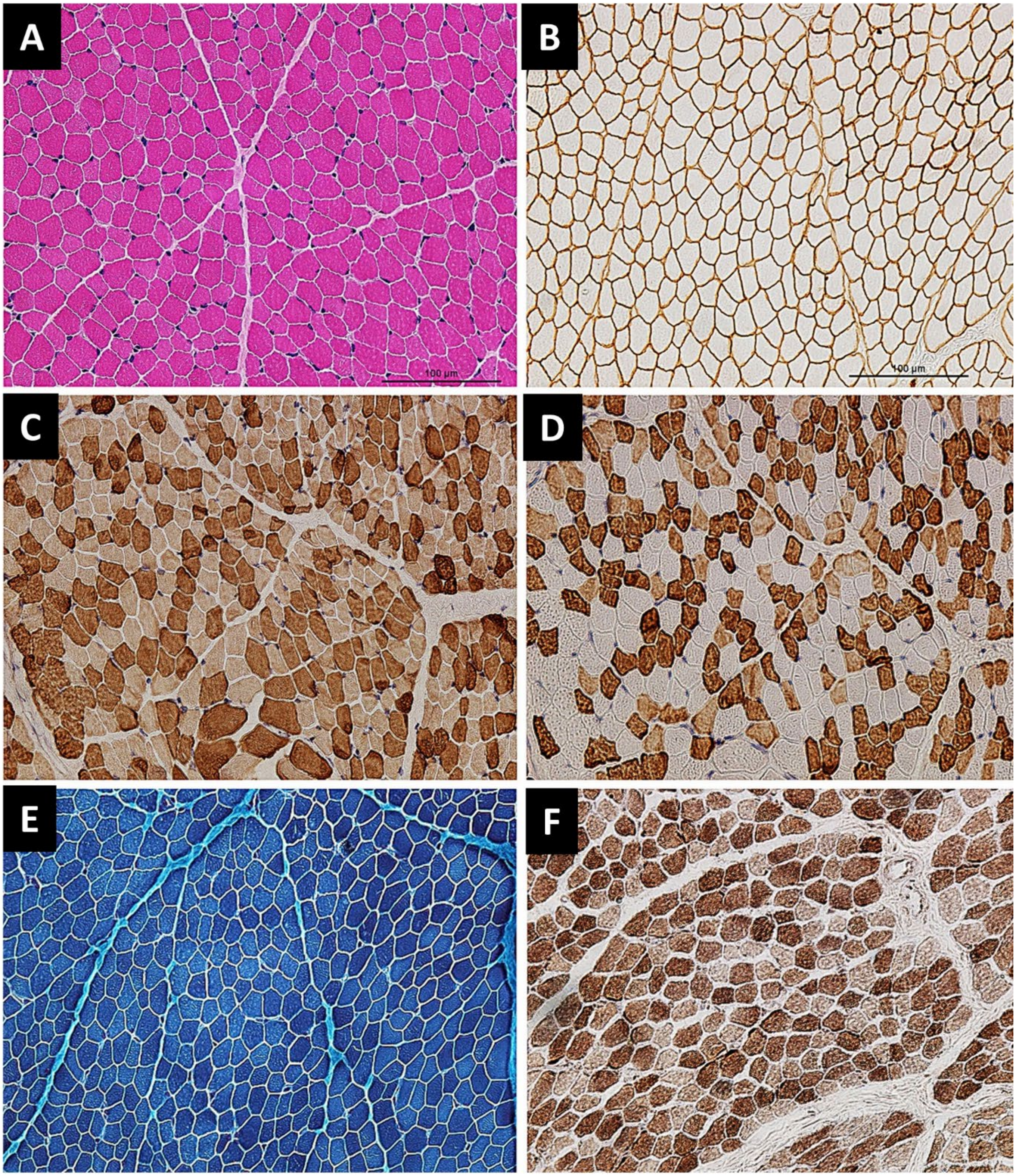

Supplement: Supplementary file 1 [file Data_Sheet_1.PDF]
